# President’s Annual Address before the California State Homœopathic Medical Society

**Published:** 1884-08

**Authors:** 


PRESIDENT'S ANNUAL ADDRESS BEFORE THE CALIFORNIA STATE HOMŒOPATHIC MEDICAL SOCIETY, May 14th, 1884. By Professor George M. Pease, M. D.

The main purpose of Dr. Pease's address is to show the necessity for earnest work on the part of his colleagues if they would achieve grand results. To this we say, amen.

"Let us, then, be up and doing,
With a heart for any fate;
Still achieving, still pursuing,
Learn to labor and to wait."

